# A comparative study of human and zebrafish glucocorticoid receptor activities of natural and pharmaceutical steroids

**DOI:** 10.3389/fendo.2023.1235501

**Published:** 2023-08-15

**Authors:** Anna Toso, Abdelhay Boulahtouf, Aurélie Escande, Clémentine Garoche, Patrick Balaguer

**Affiliations:** ^1^ Institut de Recherche en Cancérologie de Montpellier (IRCM), Inserm U1194, Université Montpellier, Institut Régional du Cancer de Montpellier (ICM), Montpellier, France; ^2^ UMR Hydrosciences Montpellier, Université de Montpellier, Montpellier, France

**Keywords:** human GR, zebrafish GR, steroids, pharmaceuticals, reporter cell lines

## Abstract

**Introduction:**

The action of environmental steroids on the human glucocorticoid receptor (hGR) has been pointed out with the risk to impair physiological immune and metabolic processes regulated by this nuclear receptor. However, there is still a lack of mechanistic information regarding their ability to interact with GR in aquatic species.

**Methods:**

To investigate ligand activation differences between hGR and zebrafish GR (zfGR), we tested several natural and synthetic steroids using reporter cell lines expressing hGR or zfGR.

**Results and discussion:**

Almost all the glucocorticoids tested (dexamethasone, cortisol, bimedrazol, medrol, cortivazol and fluticasone) are agonists of the two receptors with similar potencies. The dissociated glucocorticoids, RU24782 and RU24858 are agonists of both zfGR and hGR but with a better potency for the latter. On the other hand, the synthetic glucocorticoid forbimenol and the mineralocorticoid aldosterone are agonist on hGR but antagonist on zfGR. The other steroids tested, androgens and progestins, are all antagonists of both GRs with equal or lower potency on zfGR than on hGR. Surprisingly, the lower efficacy and potency on zfGR of aldosterone, forbimenol and the dissociated glucocorticoids is not related to their affinity for the receptors which would suggest that it could be related to less efficacious recruitment of coactivators by zfGR compared to hGR.

## Introduction

The glucocorticoid receptor (NR3C1, GR) is a ligand-activated transcription factor belonging to the family of the nuclear receptors (NRs) (1). GR is composed of three major domains: i) an N-terminal transactivation domain (NTD); ii) a small central DNA-binding domain (DBD) and iii) a C-terminal ligand binding domain (LBD) which hosts the ligand-dependent transcriptional activation function 2 (AF-2) (2). In the absence of ligand, GR is located in the cytosol and is affiliated with a large multiprotein complex that includes heat shock protein (HSP) 90, HSP70 and immunophilins (3, 4). Upon ligand binding with the ligand, the HSP complex disassociates and the receptor translocates into the nucleus to exert its transactivating effects, where it binds as a homodimer to glucocorticoid response elements (GREs) in the promoter regions of target genes. GR can also exert transrepressing effects by binding to negative GRE (nGREs) and probably by interfering with the binding of other transcription factors (5). Moreover, GR can also exert its transrepression activity independently of the DNA binding by interacting with transcription factors such as NF-kB and AP-1, which control the genes of many mediators of inflammation and immunity (6).

Glucocorticoids (GCs) are cholesterol-derived lipophilic steroid hormones produced by adrenal glands in response to external and internal signals. The main endogenous glucocorticoid hormone produced in human is cortisol, while synthetic GCs like dexamethasone and prednisolone are used extensively both in the treatment of chronic inflammatory diseases, such as rheumatoid arthritis and asthma, and for their immunosuppressant action in preventing organ rejection post transplantation (7, 8). As beneficial effects of GCs are limited by their undesirable side effects like diabetes, osteoporosis, hypertension and skin thinnings, synthetic GCs called “dissociated glucocorticoids” have been synthetized (9). These chemicals less able to maintain hGR in a conformation able to recruit coactivators than full agonists like dexamethasone displayed limited transactivation potency but strong transrepression activity (10).

Recent studies have reported that xenobiotic substances such as metals, bisphenols, vinclozolin metabolites, organotins, polybrominated diphenyl ethers and polychlorinated biphenyls can interfere with hGR (11–15). Moreover, due to the wide use of synthetic glucocorticoids and other steroids as medicaments and their incomplete removal in discharged water systems, they have been detected in the aquatic environment (16–18).

In the last decades, the presence in the environment of substances potentially interfering with NRs has been widely investigated using different *in vitro* assays expressing human NRs (19–21). However, it has been recently underlined that extrapolation of data from mammalian pharmacology and toxicology into fish species could not be appropriate due to interspecies differences between receptors (21–26) On the other hand, the use of zebrafish as model organism for aquatic toxicology is a reliable tool due to its specific features that made its use considerably grown in the last decades in scientific research. In this regard, we have established two reporter cell lines expressing respectively hGR and zfGR with the aim of improve Endocrine disrupting chemicals (EDCs) risk assessment in water quality monitoring. In this work, we tested several natural and pharmaceutical steroids on these established cell lines with the aim of identifying possible human and zebrafish differences in the capacity of these chemicals to transactivate GR, as long as we have recently pointed out species-specificity differences in the activation or inhibition of hNRs and zfNRs in water extracts (21). The findings of the current study provide new information on the activities of the chemicals on hGR and zfGR and allow the development of new biological tools to evaluate EDC in environmental samples.

## Materials and methods

### Chemicals and materials

Cell culture materials are from Life Technologies (Cergy-Pontoise, France). Luciferin (sodium salt) was purchased from Promega (Charbonnières, France). Chemical substances used in this study are presented in **Table 1**. Methyltrienolone (R1881), dexamethasone (DEX), mifepristone (RU486), aldosterone (ALDO), pregnenolone (P5), progesterone (P4), dydrogesterone (DYD), norethindrone (NET), tibolone (TIB), spironolactone (SPI), canrenone (CAN), fluticasone propionate (FT), deacetyl cortivazol/bimedrazole (DAC), cortivazol (CVZ), methylprednisolone (MPS), cortisol (CORT), dihydrotestosterone (DHT), drospirenone (DRO) and17α-hydroxyprogesterone (17-OHP) were obtained from Sigma-Aldrich (Saint-Quentin Fallavier, France). Promegestone (R5020), forbimenol, RU24782 and RU24858 are synthetic steroids non-commercially available and kindly gifts from Sanofi, Vertolaye, France.

**Table 1:** Classification of natural and synthetic steroids tested on HMLN-hGR cells and UMLN-zfGR cells.

CLASSIFICATION	COMPOUND	MOLECULAR WEIGHT (g/mol)	CAS NUMBER	MOLECULAR FORMULA	CHEMICAL STRUCTURE
glucocorticoids	bimedrazole	488.62	4906-84-7	C_30_H_36_N_2_O_4_	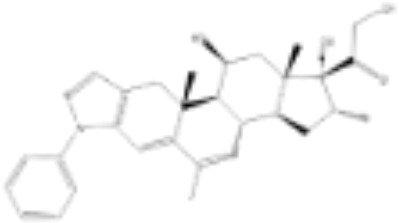
	cortisol	362.46	50-23-7	C_21_H_30_O_5_	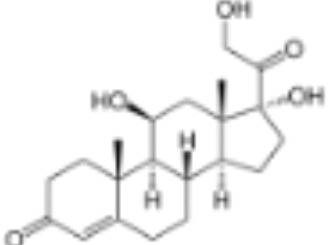
	cortivazol	530.66	1110-40-3	C_32_H_38_N_2_O_5_	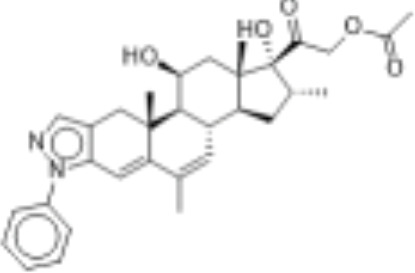
	dexamethasone	392.47	50-02-2	C_22_H_29_FO_5_	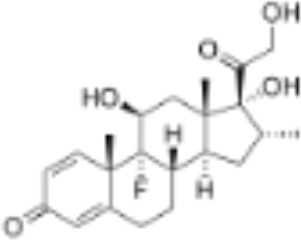
	forbimenol	490.6	–	C_27_ H_40_ O_6_	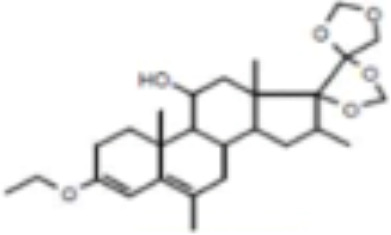
	fluticasone propionate	500.6	80474-14-2	C_25_H_31_F_3_O_5_S	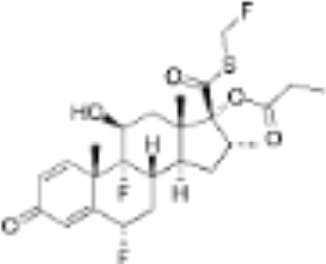
	medrol	374.47	83-43-2	C_22_H_30_O_5_	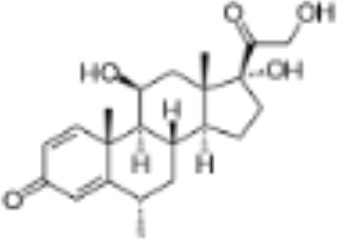
	RU24782	406.6	382-67-2	C_23_H_31_FO_3_S	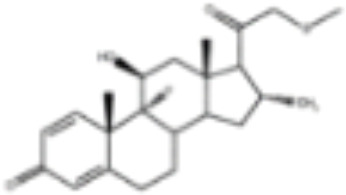
	RU24858	385.47	194413-69-9	C_23_H_28_FNO_3_	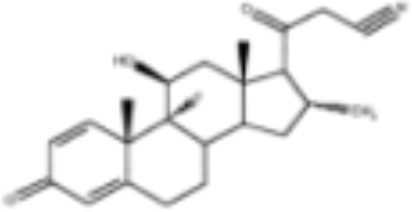
mineralocorticoids	aldosterone	360.44	52-39-1	C_21_H_28_O_5_	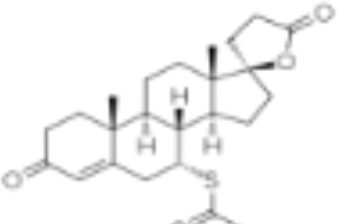
	drospirenone	366.493	67392-87-4	C_24_H_30_O_3_	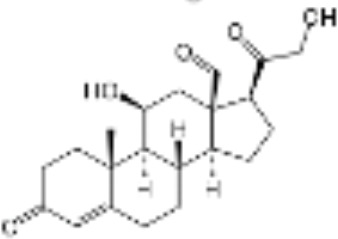
	spironolactone	416.58	52-01-7	C_24_H_32_O_4_S	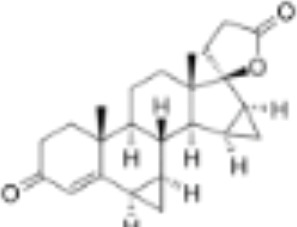
androgens	methyltrienolone	284.39	965-93-5	C_19_H_24_O_2_	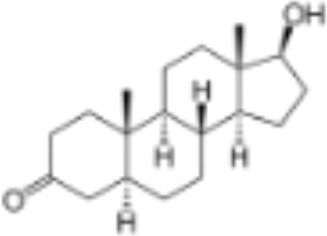
	dihydrotestosterone	290.44	521-18-6	C_19_H_30_O_2_	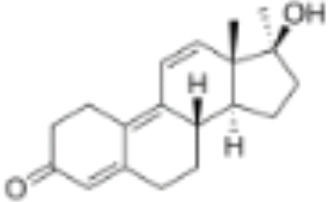
progestins	dydrogesterone	312.446	152-62-5	C_21_H_28_O_2_	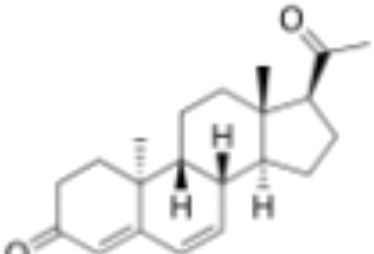
	17α-hydroxyprogesterone	330.46	68-96-2	C_21_H_30_O_3_	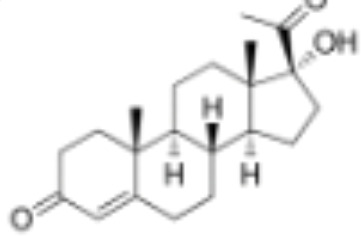
	norethindrone	298.426	68-22-4	C_20_H_26_O_2_	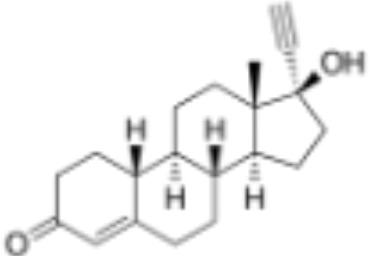
	pregnenolone	316.48	145-13-1	C_21_H_32_O_2_	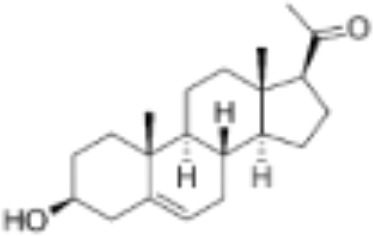
	promegestone	326.48	34184-77-5	C_22_H_30_O_2_	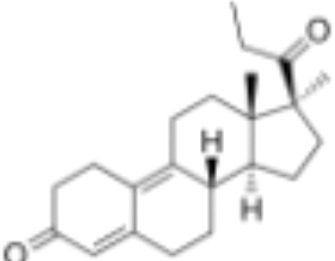
	progesterone	314.46	57-83-0	C_21_H_30_O_2_	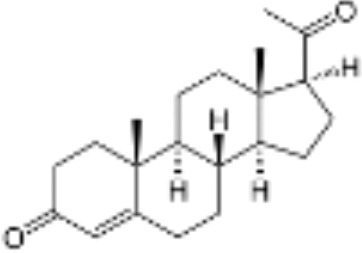
	mifepristone	429.604	84371-65-3	C_29_H_35_NO_2_	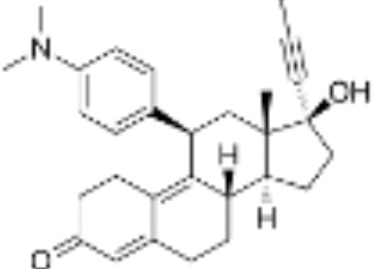

Stock solutions of chemicals were prepared in dimethyl sulfoxide (DMSO) and stored at -20°C. Fresh solution of test chemicals in test medium were prepared before each experiment. The final DMSO concentrations during treatment did not exceed 0.1% (v/v) of the test medium.

### Plasmids

MMTV-luciferase-SV-neo plasmid was already described (27). pSG5-hGR puromycin and pSG5-puromycin are kind gifts of H Gronemeyer (IGBMC, Illkirch-Graffenstaden, France). pSG5-zfGR puromycin plasmid was obtained by cloning zfGR (M1-L746) in the BamHI site of pSG5-puromycin.

### Reporter gene cell lines

HMLN-hGR and UMLN-zfGR clonal cell lines were already described (14, 21). Briefly, HMLN-hGR cells were obtained by stable co-transfection of GR positive human HeLa cells with a glucocorticoid responsive gene (MMTV-Luciferase) and a hGR (pSG5-hGR-puromycin) expressing plasmid. To obtain UMLN-zfGR cells, human U2OS cells that mildly expressed hGR (28, 29) were stably co-transfected with the MMTV-Luciferase and a zfGR expressing plasmids (pSG5-zfGR-puromycin). 48 h after the transfection, cells were treated with G418 (1 mg/ml) and puromycin (0.5 μg/ml). Within 21 days, G418 and puromycin resistant clones appeared. For each cell line, 10 clones were chosen for their ligand-induced luciferase expression. The clones were amplified, and luciferase expression was checked at several passages. For each cell line, the clone with the best induction of luciferase activity was selected and used for the screening of the different steroids. The basal expression of luciferase is 1% and 11% for HMLN-hGR and UMLN-zfGR cell lines, respectively, of the maximal luciferase expression obtained in presence of dexamethasone 100 nM. The stability and the inducibility of luciferase expression were checked during at least 20 passages (20 weeks). The stability of the hGR and zfGR expression by ligand binding assay was also checked at different passages.

To obtain UMLN-hGR pool cells, U2OS cells were stably co-transfected with the MMTV-Luciferase and the hGR expressing plasmids. ZFL-zfGR pool cells were obtained by stable co-transfection of zebrafish ZFL cells with the MMTV-Luciferase and the zfGR expressing plasmids. The transfected cells were treated 7 days with G418 and puromycin but not cloned.

HMLN- and UMLN-GR cells were grown in a 5% CO_2_ humidified atmosphere at 37°C in Dulbecco’s Modified Eagle’s Medium: Nutrient Mixture F-12 (DMEM/F-12) containing phenol red, 1 g/L glucose, 10% fetal bovine serum (FBS), 100 units/mL of penicillin, 100 µg/mL of streptomycin, 1 mg/mL geneticin and 0.5 µg/mL puromycin (culture medium). Exposure was made in phenol red-free DMEM medium supplemented with 5% of dextran-coated charcoal FBS (DCC), 100 units/mL of penicillin and 100 µg/mL of streptomycin (HMLN hGR and UMLN GR test medium).

ZFL-zfGR cells were cultured at 28°C in humidified atmosphere with 5% CO_2_ in LDF medium (50% Leibovitz 15 culture medium L15, 35% DMEM high glucose and 15% Ham’s-F12 medium) with 0.15 g/L sodium bicarbonate, 15 mM 4-(2-hydroxy-ethyl)-1-piperazineethanesulfonic acid (HEPES), 0.01 mg/mL insulin, 50 ng/mL epidermal growth factor (EGF), 50 U/mL penicillin and streptomycin antibiotics, 10% (v/v) fetal bovine serum (FBS), 1 mg/mL geneticin and 0.5 µg/mL puromycin (culture medium). Exposure was made in the same culture medium excepted that 10% (v/v) fetal bovine serum (FBS) was replaced by 5% of dextran-coated charcoal FBS (DCC-FBS). The different reporter cell lines used in this study are summarized in the **Supplementary Table 1**.

### *In vitro* transactivation assays

*In vitro* transactivation assays were performed in 96-wells white opaque clear bottom culture plates (Greiner CellStar, Dutscher, Brumath, France). GR reporter cell lines were seeded at density of 5 x 10^4^ cells per well in 150 µL culture medium and incubated at 37°C and 5% CO_2_ for 24 h. Then, medium was removed and cells were exposed to increasing dilutions of tested compounds in test medium (DMSO; final concentration 0.1% v/v). Cells were incubated for 16 h at 37°C and 5% CO_2_.

Results of transactivation activity were expressed as percentage of the maximum luciferase activity induced by dexamethasone at 10^-7^ M.

For antagonistic activity assessment, cells were exposed to different concentrations of the tested compounds and 3 nM dexamethasone. At this concentration, dexamethasone yields 60-80% of the maximal response. After the incubation period, medium was removed and replaced with 50 µL/well of test medium containing 0.3 mM luciferin. Luminescence signal was monitored in intact living cells for 2 s per well using a MicroBeta Trilux microplate scintillation and luminescence counter (PerkinElmer, Courtaboeuf, France).

The effect of the tested chemicals on cell viability was assessed with the 3-(4,5-dimethylthiazol-2-yl)-2,5-diphenyltetrazolium bromide (MTT) assay. Briefly, after luminescence detection, medium containing luciferin was removed and replaced with 100 µL/well of test medium containing 0,4 mg/ml MTT for 4 h. Colorimetric signal was monitored at 570 nM using a Pherastar microplate reader (BMG Labtech, Champigny s/Marne, france). Experiments were performed in quadruplicate and repeated three times.

### Ligand binding assays

For measurement of GR expression, HMLN-hGR, U2OS and UMLN-zfGR cells were seeded in 24-wells transparent plates at a density of 400,000 cells per well in culture medium. Cells were incubated for 24 h in a 5% CO_2_ humidified atmosphere at 37°C. Then, medium was then removed and replaced with test medium containing 10 nM [3H]-dexamethasone (84 Cu/mmol, Perkin Elmer), in the absence or presence of 10 μM of non-radioactive dexamethasone. After 3 h, unbound material was aspirated, and cells were washed three times with cold PBS in order to remove additional unbound material. Then, 0.4 ml of lysis buffer (250 mM Tris phosphate pH 7.8, 0.1% triton X-100) was added and plates were shaked for 5 min. 0.1 ml of the total cell lysate was mixed with of 0.1 ml of LSC-cocktail (Emulsifier-Safe, Perkin Elmer) and [3H] bound radioactivity was liquid scintillation counted (MicroBeta trilux, PerkinElmer). Protein concentrations of 0.1 ml of the total cell lysate were measured by Bio-Rad protein assay (Bio-Rad, Marnes-la-Coquette, France) and used to normalize bound radioactivity values expressed in dpm. Specific binding was determined by subtracting non-specific binding from total binding and enable to determine GR expression in fentomoles of protein per mg of protein. Experiments were performed in quadruplicate and repeated three times.

For ligand competition assays, HMLN-hGR and UMLN-zfGR cells were seeded in 96-wells white opaque clear bottom culture plates (Greiner CellStar, Dutscher) at density of 10^5^ cells per well in 0.2 ml of culture medium and incubated at 37°C and 5% CO_2_ for 24 h. Then, medium was removed and cells were exposed to with 1 nM [3H]-dexamethasone in the absence or presence of increasing concentrations of non-radioactive competitive compounds. After 3 h, unbound material was aspirated, and cells were washed three times with cold PBS in order to remove additional unbound material. Then, 150 μl of lysis buffer (250 mM Tris phosphate pH 7.8, 0.1% triton X-100) was added and plates were shaked for 5 min. 50 μl of the total cell lysate was mixed with of 50 μl of LSC-cocktail (Emulsifier-Safe, Perkin Elmer) and [3H] bound radioactivity was liquid scintillation counted (MicroBeta trilux, Perkin Elmer). Protein concentration of 50 μl of the total cell lysate was measured by Bio-Rad protein assay and used to normalize bound radioactivity values expressed in dpm. Results were plotted as measured dpm versus concentration of tested compound. IC50 values were defined as compound concentration required to decrease maximum [3H]-dexamethasone binding by 50%. All the experiments were performed in quadruplicates and in at least three independent experiments.

### Data analysis

Results of transactivation activity were expressed as percentage of the maximum luciferase activity induced by dexamethasone at 10^-7^ M. Dose-response curves were fitted using the sigmoidal dose-response function of a graphics and statistics software program (GraphPad Prism 8, GraphPad Software Inc.). We assumed a compound being agonist when an effect above 10% was noted as compared to the control cells, and antagonist when it decreased luciferase activity by more than 20% in the presence of the reference chemical at the concentration inducing 80% of the maximal response. Effective concentrations and inhibitory concentrations were derived from the Hill equation. For a given chemical, EC_50_ was defined as the concentration inducing 50% of its maximal effect and IC_50_ represented the concentration required for 50% inhibition. Lower and upper 95% confidence limits of EC_50_s of IC_50_s were calculated.

For ligand binding assays, dose-response curves were also fitted using GraphPad Prism. Inhibitory concentrations were derived from the Hill equation. Lower and upper 95% confidence limits of EC_50_s of IC_50_s were calculated.

## Results

### GR expression of HMLN-hGR and UMLN-zfGR reporter cell lines

**Figure 1:**
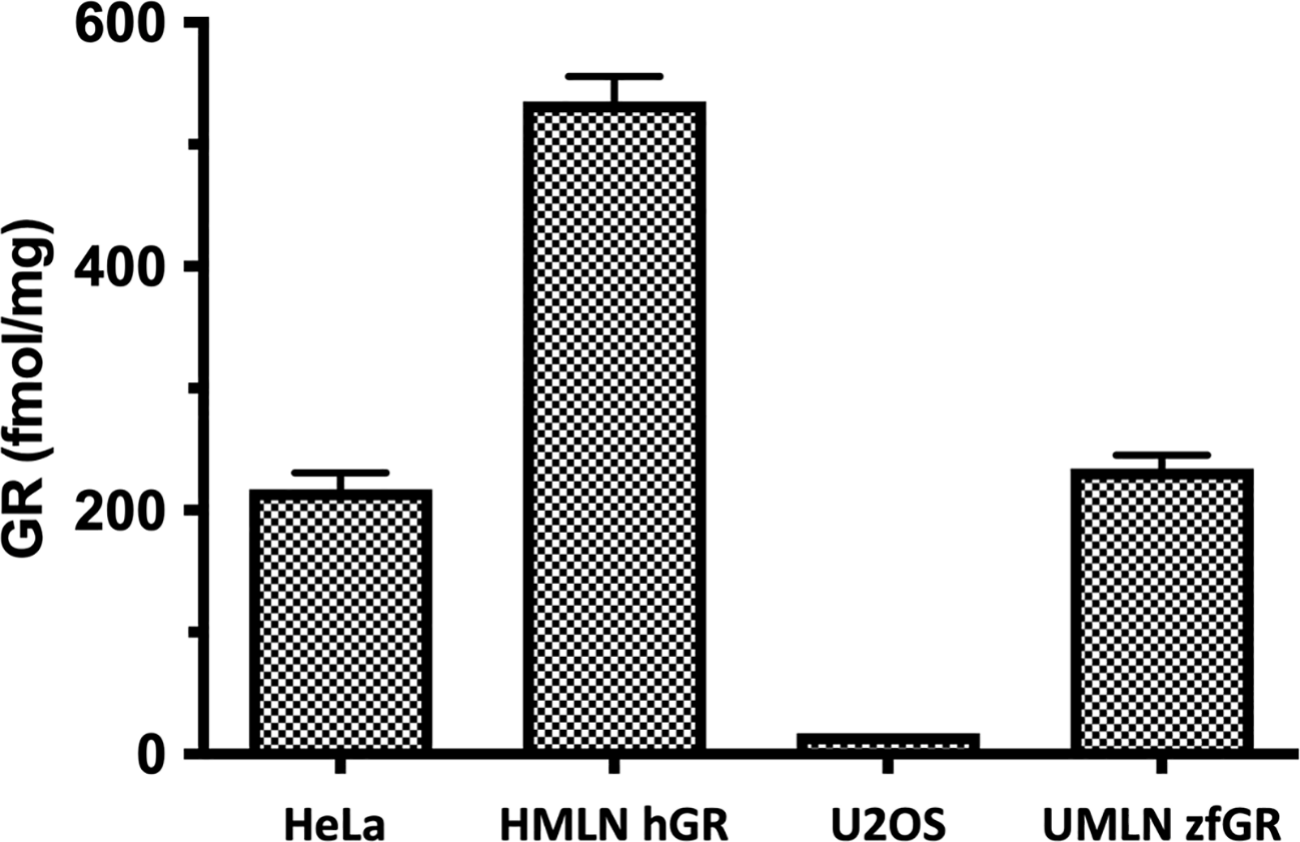
Expression of GR in HeLa, HMLN-hGR, U2OS and UMLN-zfGR cells. GR expression was measured by [3H]-DEX binding assay. Concentrations of GR are expressed in fentomoles/mg of protein. Values are means ± SD of 3 independent experiments.

The HMLN-hGR and UMLN-zfGR clonal cell lines were previously established in our laboratory (21). To obtain HMLN-hGR cells, we transfected HeLa cells which express endogenously hGR by a hGR expressing plasmid to overexpress hGR and the MMTV-Luc GR-responsive gene. The UMLN-zfGR cell line was established in U2OS cells which slightly express hGR (28, 29) by co-transfection of a zfGR expressing plasmid and the MMTV-Luc plasmid. As shown in **Figure 1**, receptor protein level (hGR or zfGR) expressed in each cell line was estimated by saturation ligand- binding assay (LBA) with 10 nM [3H]-DEX in a ‘‘whole-cell’’ experiment. These whole-cell LBAs confirmed the endogenous expression of GR in HeLa cells (217 fentomoles/mg protein) whereas in U2OS this receptor is very slightly expressed (17 fentomoles/mg protein). Stable transfection of an hGR expression plasmid enabled to increase the expression of hGR in HMLN-hGR cells (535 fentomoles/mg protein). Finally, transfection of an zfGR expression in UMLN-zfGR cells enabled to express 324 fentomoles of zfGR per mg of protein (**Figure 1**; **Supplementary Table 2**).

### hGR and zfGR transactivation by natural and pharmaceutical steroids

**Figure 2:**
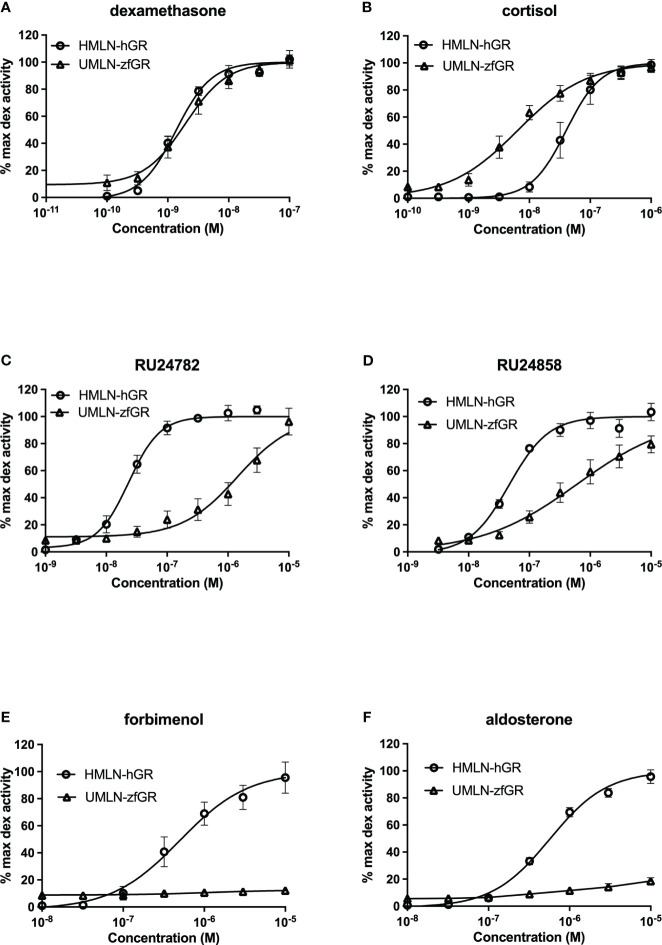
Dose-response curves of dexamethasone **(A)**, cortisol **(B)**, RU24782 **(C)**, RU24858 **(D)**, forbimenol **(E)** and aldosterone **(F)** activity in HMLN-hGR and UMLN-zfGR cells. Results are expressed as the percentage of the maximum luciferase activity induced by 100 nM dexamethasone. Error bars represent standard deviation.

The reference glucocorticoid agonist dexamethasone was tested in transactivation assays in both reporter cell models revealing close potency between the HMLN-hGR and UMLN-zfGR cell lines (EC_50_ of 1.38 and 2 nM for hGR and zfGR, respectively) (**Table 2**; **Figure 2A**). Almost all the chemicals belonging to the group of GCs, presented in **Table 1**, showed full agonistic activity both on hGR and on zfGR in transactivation assays. Bimedrazole, cortivazol, fluticasone propionate and medrol exhibited similar potency on the two nuclear receptors (**Table 2**) whereas cortisol was slighly more potent on zfGR than on hGR with EC_50_ of 40 nM and 15 nM for hGR and zfGR, respectively (**Figure 2B**; **Table 2**). On the contrary, the dissociated glucocorticoids RU24782 and RU24858 which were structurally designed to distinguish between the transrepression and transactivation (10) have showed better potency on hGR than zfGR (**Table 2**; **Figures 2C, D**). Interestingly, the synthetic glucocorticoid forbimenol only activated hGR (EC50 503 nM) (**Table 2**; **Figure 2E**). Similarly, the mineralocorticoid aldosterone, which is produced naturally in human but not in fish (30), was fully agonist on hGR but partial agonist on zfGR (maximal activity of 96 and 19% for hGR and zfGR respectively) (**Table 2**; **Figure 2F**). All the other chemicals tested lacked glucocorticoid agonist activity.

**Table 2:** Agonistic activity assessment.

	HMLN-hGR				UMLN-zfGR			
Ligands	EC50 (nM)	lower and upper 95% conf. limit (nM)	% max act	REP	EC50 (nM)	lower and upper 95% conf. limit (nM)	% max act	REP
DMSO			1 ± 0.2				11 ± 2	
bimedrazole	0.04	0.04 to 0.05	104 ± 7	32	0.05	0.04 to 0.06	100 ± 6	37.8
cortivazol	0.18	0.14 to 0.24	97 ± 6	7.8	0.38	0.32 to 0.43	95 ± 6	5.2
dexamethasone	1.4	1 to 1.8	100 ± 0	1	2	1.4 to 2.6	100 ± 0	1
fluticasone proprionate	0.22	0.1 to 0.5	119 ± 9	6.2	0.23	0.16 to 0.32	95 ± 4	8.6
medrol	8.4	4.4 to 15.6	119 ± 5	0.16	12	10 to 15	104 ± 4	0.16
cortisol	40	35 to 45	99 ± 4	0.035	15	9 to 27	93 ± 4	0.131
RU24782	21	6 to 72	130 ± 7	0.07	1504	888 to 2546	96 ± 10	0.001
RU24858	46	31 to 69	103 ± 6	0.03	743	433 to 1274	79 ± 6	0.003
forbimenol	498	360 to 690	96 ± 12	0.003	–	–	–	–
aldosterone	567	449 to 716	96 ± 5	0.002	1243	802 to 1865	19 ± 3	0.002
mifepristone	6.7	5.4 to 8.3	12 ± 1	0.205	10	8 to 12	30 ± 5	0.198

EC_50_s are expressed in nM. Values of EC_50_ are the mean from at least three separate experiments. Lower and upper 95% confidence limits of EC_50_s are indicated. Maximal activities (% max act) of the chemicals tested for their agonistic activity are expressed as a percentage of the maximal luciferase activity induced by 100 nM dexamethasone. They were determined at 10^-5^M excepted for bimedrazole, cortivazol and dexamethasone. For these chemicals, the maximal concentration tested was 10^-6^M. Relative potency (REP) of each competitor was calculated as ratio of concentrations of DEX or chemical required to induce the specific transactivation by 50% (ratio of EC50 values). REP value for DEX was arbitrarily set at 1.

Half maximal effective concentration (EC_50_), maximal activity (% max act) and relative potency (REP) of the chemicals on hGR and zfGR.

**Figure 3:**
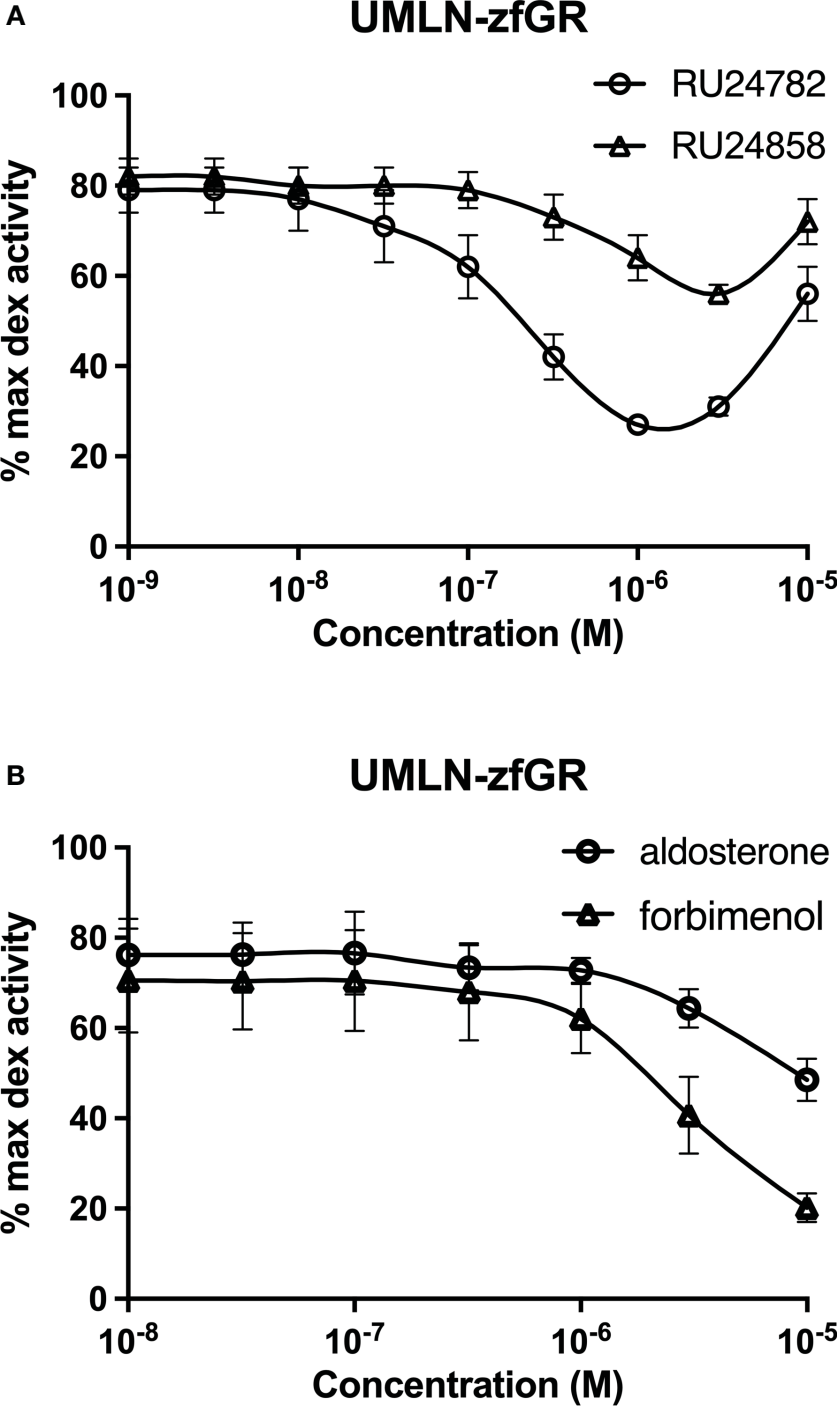
Dose-response curves of RU24782 and RU24858 **(A)**, aldosterone and forbimenol **(B)** activity in UMLN-zfGR cells in presence of dexamethasone 3 nM. Results are expressed as percentage of the maximum luciferase activity induced by 100 nM M dexamethasone. Error bars represent standard deviation.

**Figure 4:**
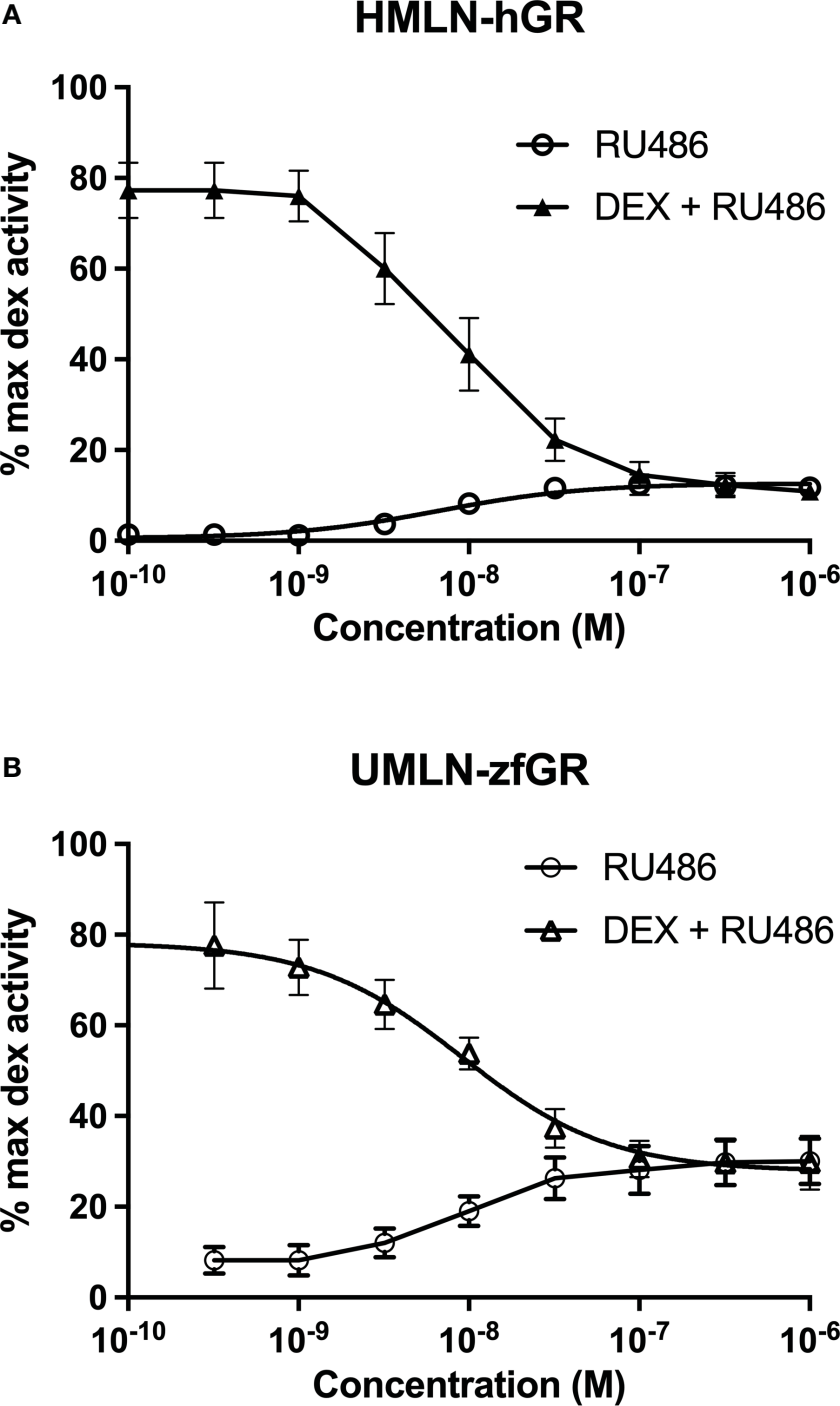
Dose-response curves of mifepristone activity in HMLN-hGR **(A)** and UMLN-zfGR **(B)** cells in absence or presence of dexamethasone 3 nM. Results are expressed as percentage of the maximum luciferase activity induced by 100 nM dexamethasone. Error bars represent standard deviation.

RU24782, RU24858, forbimenol and aldosterone were also assessed for antagonism in UMLN-zfGR cells. Interestingly, RU24782, RU24858 partially repress dexamethasone-induced activity at sub-micromolar concentrations (**Figure 3A**). As these GCs were described as less able to recruit coactivators than dexamethasone to hGR (10), their zfGR antagonism at sub-micromolar concentrations and agonism at micromolar concentrations is probably relied to their lower efficacy for recruiting coactivators. Forbimenol and aldosterone also antagonized zfGR (IC50 3160 and 13980 nM, respectively) (**Table 3**; **Figure 3B**). Finally, the anti-progestin mifepristone was partial agonist of hGR and zfGR with higher efficacy on the last one (**Table 2**; **Figures 4A, B**). In presence of dexamethasone, it acts as partial antagonist with IC50 in the 10 to 40 nanomolar range (**Table 3**; **Figures 4A, B**). The other chemicals lacking glucocorticoid agonist activity were also tested for their antagonist activity. They all exhibited antagonist activity on hGR and zfGR. IC50 varied between 772 (promegestone) and 6101 (dihydrostestosterone) nM for hGR and 1066 (17α-hydroxyprogesterone) and 7711 (spironolactone) nM for zfGR (**Table 3**).

**Table 3:** Antagonistic activity assessment.

	HMLN-hGR			UMLN-zfGR		
Ligands	IC50 (nM)	lower and upper 95% conf. limit (nM)	% min act	IC50 (nM)	lower and upper 95% conf. limit (nM)	% min act
mifepristone	12	6 to 23	11 ± 2	37	10 to 128	30 ± 6
forbimenol	–	–	–	3160	2134 to 4670	20 ± 4
aldosterone	–	–	–	13980	12440 to 15710	49 ± 5
drospirenone	1128	562 to 2265	2 ± 1	1510	1037 to 2200	12 ± 4
spironolactone	5298	1608 to 17450	11 ± 4	7711	4239 to 14030	32 ± 7
methyltrienolone	1493	640 to 3480	2 ± 1	2760	1549 to 4918	19 ± 7
dihydrotestosterone	6101	3549 to 10490	26 ± 7	nc	nc	nc
promegestone	772	453 to 1315	3 ± 1	1108	848 to 1448	8 ± 2
norethindrone	1064	580 to 1953	2 ± 1	2794	1493 to 5229	16 ± 4
17α-hydroxyprogesterone	1719	1013 to 2916	14 ± 4	1066	733 to 1549	18 ± 1
progesterone	2264	476 to 10770	4 ± 1	1232	780 to 1945	10 ± 1
dydrogesterone	2704	1341 to 5450	6 ± 1	3982	3019 to 5252	24 ± 6
pregnenolone	6032	3216 to 11310	22 ± 4	nc	nc	nc

IC_50_s are expressed in nM. Values of IC_50_ are the mean from at least three separate experiments. Lower and upper 95% confidence limits of IC_50_s are indicated. Minimal activities (% min act) of the chemicals tested for their antagonistic activity are expressed as a percentage of the maximal luciferase activity induced by 100 nM dexamethasone. They were determined at 10^-5^M. nc not calculated.

Half maximal inhibitory concentration (IC_50_) and minimal activity of the chemicals on hGR and zfGR.

### Differences between hGR and zfGR transactivation are not due to differences of affinity

Whole-cell competitive binding assays were performed with HMLN-hGR and UMLN-zfGR cells to determine whether the different potencies observed in transactivation assays for some of the chemicals reflected their abilities to differently bind to hGR and zfGR. **Table 4** summarized IC_50_ and RBA values for hGR and zfGR. IC_50_ of DEX of 5.8 and 3.1 nM for hGR and zfGR, respectively confirmed the similar potency of this chemical for the two receptors. By contrast, cortisol bound preferentially zfGR as shown by IC_50_s of 55.2 and 3.5 nM for hGR and zfGR, respectively (**Table 4**) and thus explained its higher potency on the fish receptor. RU24782 bound preferentially to hGR than to zfGR but these differences in affinity did not reflect the differences in potency reinforcing the hypothesis that that this chemical is less able to recruit coactivators for zfGR than for hGR. Finally, aldosterone and forbimenol which are agonists on hGR and antagonists on zfGR bound also slightly preferentially hGR than zfGR but these differences in affinity do not explain their differences in activities for the two receptors.

**Table 4:** Whole-cell ligand competition assays.

	HMLN-hGR			UMLN-zfGR		
Ligands	IC50 (nM)	lower and upper 95% conf. limit (nM)	RBA	IC50 (nM)	lower and upper 95% conf. limit (nM)	RBA
dexamethasone	5.8	4.5 to 7.4	100	3.1	2.4 to 4	100
cortisol	55.2	43.8 to 71.1	3.5	14.7	10.3 to 17.2	13.1
RU24782	44.3	34.4 to 57.1	13.1	108.9	72.6 to 165	28.5
forbimenol	409	192 to 891	1.41	539	403 to 1720	0.58
aldosterone	794	471 to 1350	0.073	1368	861 to 2180	0.226

Whole-cell ligand competition assays were performed using 1 nM [3H]-DEX as tracer. IC_50_s are expressed in nM. Values of IC_50_ are the mean from at least three separate experiments. Lower and upper 95% confidence limits of IC_50_s are indicated. RBA of each competitor was calculated as ratio of DEX or chemical concentration required to reduce the specific radioligand binding by 50%. RBA is relative binding affinity where DEX = 100.

Half minimal effective concentration (IC50) and relative binding activity (RBA) of DEX, cortisol, RU24782, forbimenol and aldosterone on hGR and zfGR.

### Differences between hGR and zfGR transactivation are not dependant of the cellular context

**Figure 5:**
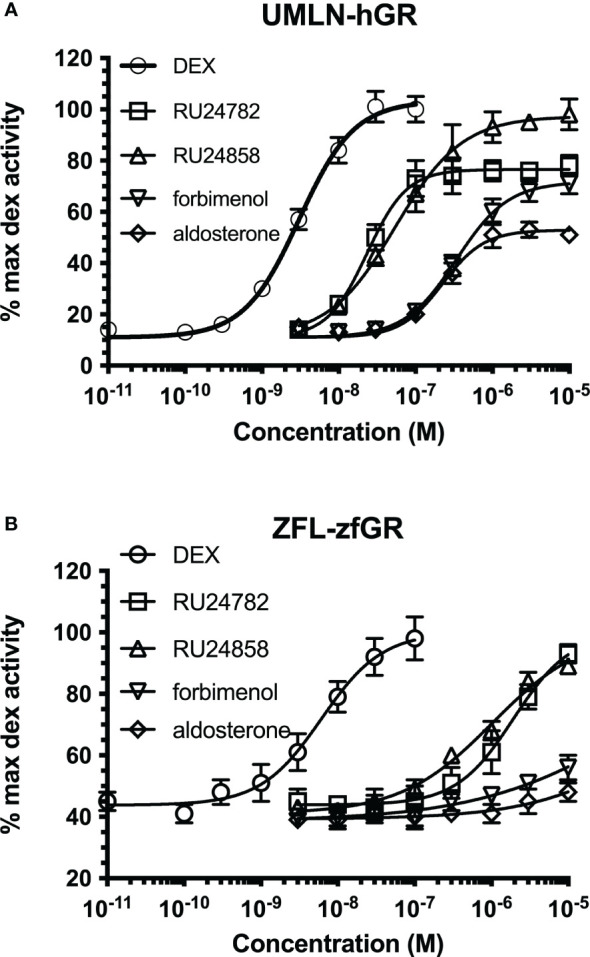
Dose-response curves of dexamethasone, RU24782, RU24858, aldosterone and forbimenol activity in UMLN-hGR **(A)** and ZFL-zfGR **(B)** cells. Results are expressed as percentage of the maximum luciferase activity induced by 100 nM dexamethasone. Error bars represent standard deviation.

Since cellular context could influence the transcriptional activity of NRs and hGR and zfGR reporter cell lines are different (Hela for hGR and U2OS for zfGR), we established UMLN-hGR pool cells. We tested in these cells the activity of RU24782, RU24858, forbimenol and aldosterone. The response profiles obtained (**Figure 5A**) are very similar to those obtained with the HMLN-hGR cell line indicating that at least for hGR these chemicals had a similar potency. The four chemicals were agonists on these cells and the EC_50_ values for RU24782, RU24858, forbimenol and aldosterone were respectively of 3.1, 23.3, 54.5, 338 and 224 nM.

We also tested in zebrafish liver ZFL-zfGR pool cells the activity of the three chemicals. Again, the response profiles obtained (**Figure 5B**) are very similar to those obtained with the UMLN-zfGR cell line. The EC_50_ value for DEX, RU24782 and RU24858 were respectively of 6, 1938 and 1027 nM were respectively whereas forbimenol and aldosterone only slightly activated luciferase expression (**Figure 5B**).

## Discussion

Previous studies investigating progestin receptor (PR) and mineralocorticoid (MR) receptor have showed strong differences in binding affinity and transactivation properties of steroids (24, 31). As few studies have been done on GR, in this work, we have evaluated the ability of 21 steroids to alter the transcriptional activity of hGR and zfGR.

Our data have showed that the majority of the steroids tested bind with a similar affinity to both GR. However, some of them presented marked differences in transactivation efficacies and potencies. The most different steroids are forbimenol and aldosterone. These chemicals have a full agonist profile on hGR while they do not induce luciferase activity in UMLN-zfGR cells. According to their ability to bind to zfGR, aldosterone and forbimenol were able to antagonize DEX-induced luciferase activity in a concentration-dependant manner in the zfGR assay. Strikingly, the dissociated glucocorticoids RU24782 and RU24858 which are synthetic GCs less able to recruit coactivators than full agonists (dexamethasone, bimedrazol) to hGR have similar affinities for hGR and zfGR but showed different potencies on the two receptors. Even more curiously, these compounds are zfGR antagonists at low concentrations (at which they bind to zfGR) and zfGR agonists at high concentrations.

As these four steroids have similar affinities for both GRs, their zfGR antagonism is probably relied to their lower efficacy for recruiting coactivators. Interestingly, we observed the same results in different cellular contexts (U2OS for hGR and ZFL for zfGR); indicating that these differences are probably not specific to the cellular context, but on the contrary reflected the intrinsic properties of steroids on zfGR.

Although we did not fully explain the differences of activity of the two GRs, we could point out that these differences could probably be due to different coactivators’ recruitment by hGR and zfGR. This should be further confirmed by using site-directed mutagenesis and analyses of the structures of the hGR and zfGR ligand binding domain which are currently lacking. These techniques have made it possible to explain that progesterone and spironolactone act as antagonists on hMR and agonists on zfMR due to a substitution of threonine in hMR by a leucine on zfMR (32). These techniques would be probably also valuable to explain why several progestins are antagonist on hPR while they are agonist on hPR (24).

Although few of the steroids tested exhibited differences in activation between human and zebrafish receptor for GR than for MR and PR, our results showed that they exist and confirm that investigating inter-species differences is important and can improve EDC risk assessment. According to our results, toxicological data extrapolated from mammalian models could not be always suitable for predicting possible hazard in other species such as zebrafish. Regarding this latter aspect, since in the last decades numerous studies have detected glucocorticoid activity in the environment raising concern about their distribution and their harmful effects on wildlife (16; 33), we consider that the use of our established cell lines could improve the endocrine disrupting assessment and environmental contamination monitoring.

## Data availability statement

The original contributions presented in the study are included in the article/**Supplementary Material**. Further inquiries can be directed to the corresponding author.

## Ethics statement

For reporter cell lines experiments (luciferase expression experiments, binding analysis), ethical approval was not required. The studies were conducted in accordance with local legislation and institutional requirements. The human samples used in this study were acquired from another research group. Written informed consent to participate in this study was not required from the participants or the participants’ legal guardians/next of kin in accordance with the national legislation and the institutional requirements.

## Author contributions

AT and AB performed experiments. AT and PB conceived the work. AT drafted the original manuscript and prepared the figures and tables. AE, CG and PB revised the manuscript. PB supervised the work. All authors contributed to the article and approved the submitted version.

## References

[1] Lu NZ Wardell SE Burnstein KL Defranco D Fuller PJ Giguere V. International Union of Pharmacology. LXV. The pharmacology and classification of the nuclear receptor superfamily: glucocorticoid, mineralocorticoid, progesterone, and androgen receptors. Pharmacol Rev (2006) 58:782. doi: 10.1124/pr.58.4.9 17132855

[2] Kumar R Thompson EB. Gene regulation by the glucocorticoid receptor: structure: function relationship. J Steroid Biochem Mol Biol (2005) 94:383. doi: 10.1016/j.jsbmb.2004.12.046 15876404

[3] Noddings CM Wang RY Johnson JL Agard DA. Structure of Hsp90-p23-GR reveals the Hsp90 client-remodelling mechanism. Nature (2022) 601:465. doi: 10.1038/s41586-021-04236-1 34937936 PMC8994517

[4] Wang RY Noddings CM Kirschke E Myasnikov AG Johnson JL Agard DA. Structure of Hsp90-Hsp70-Hop-GR reveals the Hsp90 client-loading mechanism. Nature (2022) 601:460. doi: 10.1038/s41586-021-04252-1 34937942 PMC9179170

[5] Santos GM Fairall L Schwabe JW. Negative regulation by nuclear receptors: a plethora of mechanisms. Trends Endocrinol Metab (2011) 22:87. doi: 10.1016/j.tem.2010.11.004 21196123 PMC3053446

[6] Kadmiel M Cidowski JA. Glucocorticoid receptor signaling in health and disease. Trends Pharmacol Sci (2013) 34:518. doi: 10.1016/j.tips.2013.07.003 23953592 PMC3951203

[7] Baschant U Tuckermann J. The role of the glucocorticoid receptor in inflammation and immunity. J Steroid Biochem Mol Biol (2010) 120:69. doi: 10.1016/j.jsbmb.2010.03.058 20346397

[8] Oakley RH Cidlowski JA. The biology of the glucocorticoid receptor: new signaling mechanisms in health and disease. J Allergy Clin Immunol (2013) 132:1033. doi: 10.1016/j.jaci.2013.09.007 24084075 PMC4084612

[9] Vayssière BM Dupont S Choquart A Petit F Garcia T Marchandeau C. Synthetic glucocorticoids that dissociate transactivation and AP-1 transrepression exhibit antiinflamatory activity *in vivo* . Mol Endocrinol (1997) 11:1245. doi: 10.1210/mend.11.9.9979 9259316

[10] Dezitter X Fagart J Taront S Fay M Masselot B Hétuin D. A structural explanation of the effects of dissociated glucocorticoids on glucocorticoid receptor transactivation. Mol Pharmacol (2014) 85:226. doi: 10.1124/mol.113.085860 24225022

[11] Molina-Molina JM Hillenweck A Jouanin I Zalko D Cravedi JP Fernández M. Steroid receptor profiling of vinclozolin and its primary metabolites. Toxicol Appl Pharmacol (2006) 216:44. doi: 10.1016/j.taap.2006.04.005 16750840

[12] Gumy C Chandsawangbhuwana C Dzyakanchuk AA Kratschmar DV Baker ME Odermatt A. Dibutyltin disrupts glucocorticoid receptor function and impairs glucocorticoid-induced suppression of cytokine production. PloS One (2008) 3:e3545. doi: 10.1371/journal.pone.0003545 18958157 PMC2568824

[13] Gulliver LSM. Xenobiotics and the glucocorticoid receptor. Toxicol Appl Pharmacol (2017) 319:69. doi: 10.1007/978-1-4939-9195-2_4 28174120

[14] Grimaldi M Boulahtouf A Toporova L Balaguer P. Functional profiling of bisphenols for nuclear receptors. Toxicology (2019) 420:39. doi: 10.1016/j.tox.2019.04.003 30951782

[15] Zhang J Yang Y Liu W Schlenk D Liu J. Glucocorticoid and mineralocorticoid receptors and corticosteroid homeostasis are potential targets for endocrine-disrupting chemicals. Environ Int (2019) 133(Pt A):105133. doi: 10.1016/j.envint.2019.105133 31520960

[16] Weizel A Schlüsener MP Dierkes G Ternes TA. Occurrence of glucocorticoids, mineralocorticoids, and progestogens in various treated wastewater, rivers, and streams. Environ Sci Technol (2018) 52:5296. doi: 10.1021/acs.est.7b06147 29580053

[17] Hashmi MAK Krauss M Escher BI Teodorovic I Brack W. Effect-directed analysis of progestogens and glucocorticoids at trace concentrations in river water. Environ Toxicol Chem (2020) 39:189. doi: 10.1016/j.scitotenv.2017.12.187 31614391

[18] Shen X Chang H Sun Y Wan Y. Determination and occurrence of natural and synthetic glucocorticoids in surface waters. Environ Int (2020) 134:105278. doi: 10.1016/j.envint.2019.105278 31711021

[19] Leusch FDL Neale PA Arnal C Aneck-Hahn NH Balaguer P Bruchet A. Analysis of endocrine activity in drinking water, surface water and treated wastewater from six countries. Water Res (2018) 139:10. doi: 10.1016/j.watres.2018.03.056 29621713

[20] Alygizakis NA Besselink H Paulus GK Oswald P Hornstra LM Oswaldova M. Characterization of wastewater effluents in the Danube River Basin with chemical screening, *in vitro* bioassays and antibiotic resistant genes analysis. Environ Int (2019) 127:420. doi: 10.1016/j.envint.2019.03.060 30959307

[21] Neale PA Grimaldi M Boulahtouf A Leusch FDL Balaguer P. Assessing species-specific differences for nuclear receptor activation for environmental water extracts. Water Res (2020) 185:116247. doi: 10.1016/j.watres.2020.116247 32758789

[22] Pinto C Grimaldi M Boulahtouf A Pakdel F Brion F Cavaillès V. Selectivity of natural and environmental estrogens for zebrafish estrogen receptors. Toxicol Appl Pharmacol (2014) 280:29. doi: 10.1016/j.taap.2014.07.020 PMC8740820 25106122

[23] Pinto C Hao R Grimaldi M Thrikawala S Boulahtouf A Aït-Aïssa S. Differential activity of BPA, BPAF and BPC on zebrafish estrogens receptors *in vitro* and *in vivo* . Toxicol Appl Pharmacol (2019) 380:114709. doi: 10.1016/j.taap.2019.114709 31415773 PMC6748385

[24] Garoche C Aït-Aïssa S Boulahtouf A Creusot N Hinfray N Bourguet W. Human and zebrafish nuclear progesterone receptors are differently activated by manifold progestins. Environ Sci Technol (2020) 54:9510. doi: 10.1021/acs.est.0c02056 32650635

[25] Creusot N Garoche C Grimaldi M Boulahtouf A Chiavarina B Bourguet W. A Comparative study of uman and zebrafish pregnane X receptor activities of pesticides and steroids using *in vitro* reporter gene assays. Front Endocrinol (Lausanne) (2021) 12:665521. doi: 10.3389/fendo.2021.665521 34084152 PMC8167039

[26] Garoche C Boulahtouf A Grimaldi M Chiavarina B Toporova L den Broeder MJ. Interspecies differences in activation of peroxisome proliferator-activated receptor γ by pharmaceutical and environmental chemicals. Environ Sci Technol (2021) 55:16489. doi: 10.1021/acs.est.1c04318 34843233

[27] Térouanne B Tahiri B Georget V Belon C Poujol N Avances C. A stable prostatic bioluminescent cell line to investigate androgen and antiandrogen effects. Mol Cell Endocrinol (2000) 160:39. doi: 10.1016/s0303-7207(99)00251-8 10715537

[28] Sonneveld E Jansen HJ Riteco JA Brouwer A van der Burg B. Development of androgen- and estrogen-responsive bioassays, members of a panel of human cell line-based highly selective steroid-responsive bioassays. Toxicol Sci (2005) 83:136. doi: 10.1093/toxsci/kfi005 15483189

[29] Milcamps A Liska R Langezaal I Casey W Dent M Odum J. Reliability of the AR-CALUX®. *In vitro* method used to detect chemicals with (anti)androgen activity: results of an international ring trial. Toxicol Sci (2021) 184:170. doi: 10.1093/humupd/7.3.248 34165557 PMC8557474

[30] Cruz SA Lin CH Chao PL Hwang P. Glucocorticoid receptor, but not mineralocorticoid receptor, mediates cortisol regulation of epidermal ionocyte development and ion transport in zebrafish (*Danio rerio*). PloS One (2013) 81:219. doi: 10.5935/0004-2749.20180044 PMC3812134 24205060

[31] Baker ME. Divergent evolution of progesterone and mineralocorticoid receptors in terrestrial vertebrates and fish influences endocrine disruption. Biochem Pharmacol (2022) 198:114951. doi: 10.1016/j.bcp.2022.114951 35149051

[32] Fuller PJ Yao YZ Jin R He S Martín-Fernández B Young MJ. Molecular evolution of the switch for progesterone and spironolactone from mineralocorticoid receptor agonist to antagonist. Proc Natl Acad Sci U S A. (2019) 116:18578. doi: 10.1073/pnas.1903172116 31439819 PMC6744879

[33] Odermatt A Gumy C Atanasov AG Dzyakanchuk AA. Disruption of glucocorticoid action by environmental chemicals: potential mechanisms and relevance. J Steroid Biochem Mol Biol (2006) 102:222. doi: 10.1016/j.jsbmb.2006.09.010 17045799

