# Supplementary material for: A comparative study of human and zebrafish glucocorticoid receptor activities of natural and pharmaceutical steroids
Source: Front Endocrinol (Lausanne). 2023 Aug 15;14:1235501. doi: 10.3389/fendo.2023.1235501 (PMC10466050; doi:10.3389/fendo.2023.1235501)
Supplement: Supplementary file 1 [file Table_1.pdf]

Supplementary Table 1. Reporter cell lines used in the study.

Name of the Reporter cell line	Cell origin	GR expressing plasmid	Luc reporter gene	Cell line state
HMLN hGR	HeLa (human)	pSG5-hGR-puromycin	MMTV-Luciferase-neomycin	Clonal
UMLN zfGR	U2OS (human)	pSG5-zfGR-puromycin	MMTV-Luciferase-neomycin	Clonal
UMLN hGR	U2OS (human)	pSG5-hGR-puromycin	MMTV-Luciferase-neomycin	Pool cell population
ZFL zfGR	ZFL (zebrafish)	pSG5-zfGR-puromycin	MMTV-Luciferase-neomycin	Pool cell population
