# Supplementary material for: A comparative study of human and zebrafish glucocorticoid receptor activities of natural and pharmaceutical steroids
Source: Front Endocrinol (Lausanne). 2023 Aug 15;14:1235501. doi: 10.3389/fendo.2023.1235501 (PMC10466050; doi:10.3389/fendo.2023.1235501)
Supplement: Supplementary file 2 [file Table_2.pdf]

Supplementary Table 2. GR expression in HeLa, HMLN-hGR, U2OS and UMLN-zfGR cells. GR expression was measured by [^{3}H]-DEX binding assays and expressed in fentomoles/mg of protein. Values are means $\pm$ SD of 3 independent experiments.

Cell line	hGR expression (fmol/mg)
HeLa	394 +/- 42
HMLN-hGR	535 +/- 30
U2OS	17 +/- 0.49
UMLN-zfGR	253 +/- 65
